# CircCASC15-miR-100-mTOR may influence the cervical cancer radioresistance

**DOI:** 10.1186/s12935-022-02573-3

**Published:** 2022-04-27

**Authors:** Tingting Yao, Yao Yao, Zhiliao Chen, Yongpai Peng, Guanglei Zhong, Chunxian Huang, Jing Li, Ruixin Li

**Affiliations:** 1 Department of Gynecological Oncology, Sun Yat-Sen Memorial Hospital, Sun Yat-Sen University, 107 Yan Jiang West Road, Guangzhou, 510120 People’s Republic of China; 2 Key Laboratory of Malignant Tumor Gene Regulation and Target Therapy of Guangdong Higher Education Institutes, Sun Yat-Sen University, Guangzhou, China; 3 Guangdong Food and Drug Vocational College, Guangzhou, China

**Keywords:** Cervical cancer, Radio-resistance, miR-100, BSP, circCASC15

## Abstract

**Background:**

Cervical cancer has ranked the top one in gynecological malignancies for incidence. Radioresistance is now becoming a leading reason of recurrence.

**Methods:**

Our microRNA array data indicated that the miRNA-100 level decreased significantly during radioresistance. In this study, we up-regulated miR-100 in Hela and Siha cells by using miR-100 mimics and observed proliferation and invasion.

**Results:**

It turned out that with overexpression of miR-100, the cells had less invasiveness as well as proliferation. It may target gene mTOR, and it deed reduced EMT. To examine the role of miR-100 in radioresistance, there was no significant result showed by BSP. While the circCASC15 has been identified with sponge function according to RNA pull down and ISH.

**Conclusion:**

The conclusions indicate miR-100 is a tumor suppressor gene and could be a therapeutic target in radio-resistant cervical cancers.

## Introduction

Cervical cancer is one of the most common malignant tumors in women, whose morbidity and mortality has ranked the fourth of female malignant tumors. In 2018, approximately 570,000 cases of cervical cancer have been diagnosed and 311,000 died of it [1]. Early stage can be managed surgically with cervical cone biopsy, simple or radical hysterectomy, with pelvic lymphadenectomy or radiotherapy. For late stage, the recommended therapeutic options also include chemotherapy, external beam radiation, and brachytherapy. So radiotherapy (RT) has been widely applied from stage I to stage IV cervical cancer as primary or postoperative adjuvant treatment, and maybe the only treatment available for many patients with advanced cervical cancer. However, recurrence after radiotherapy remains significant. Approximately 20% of patients diagnosed with pelvic recurrence, while the cure rate for early-stage is more than 80% [2]. The 5-year survival rate of recurrence is between 10.1% and 22.3% [3, 4]. Therefore, elimination of radioresistance was important for patients diagnosed with cervical cancer.

MicroRNAs (miRNAs) belong to non-coding RNA family that function at the post-transcriptional level. Their deregulated expression can influence both treatment response [5] and development of drug resistance [6, 7].MiR-100 belongs to miR-99 family, which located in chromosome 11. It has been proved playing a role in proliferation, metastasis, as well as sensitivity to radiotherapy and chemotherapy [8–11].

In human cancers, DNA hypermethylation of CPG islands in gene promoters has been reported as one mechanism for down-regulating tumor suppressor genes. Aberrant CpG methylation in miRNA promoter regions may contribute to their dysregulation in some cancers [12]. An estimated 10% of miRNAs are regulated epigenetically through DNA methylation. To further investigate whether the downregulation of MiR-100 originates from the hypermethylation of the genomic region upstream of MiR-100, Bisulfite Sequencing Polymerase Chain Reaction (BSP) was used.

Circular RNA (circRNA) is another kind of non-coding RNA because it is a closed covalent loop without poly A tail or 5'to 3' polarity [13]. At present, more than 30,000 circRNAs have been identified [14]. A variety of circRNAs are believed to have relationship with occurrence of tumors [15]. Several reports have showed that miRNA response elements exist in circRNAs, which act as competitive endogenous RNAs (ceRNAs) to sponge miRNAs or transcriptional regulators**.**

In this study, we up-regulated miR-100 and found more apoptosis and less invasiveness as well as proliferation. It may also influence cell cycle via target gene mTOR, and it deed reduced EMT. In order to detect the mechanism of miR-100 in radioresistance, BSP showed no significance. While the circCASC15 has been identified with sponge function according to RNA pull down and ISH. We found miR-100 downregulated in radioresistant cervical cancer and found circCASC15 may be the sponge of miR-100.

## Materials and methods

### Patient selection and human tissues

#### Group 1 (for miRNA real time polymerase chain reaction array)

Three patients have been clarified as stage IIB-IVA according to International Federation of Gynecology and Obstetrics (FIGO), who had received radiotherapy in our hospital between 2000 and 2008. According to the 2013 NCCN cervical cancer clinical practice guidelines, patients received standard radiotherapy, pelvic radiotherapy and branch radiotherapy, with a total dose of 70 Gy at point A [16]. Two pathologists evaluated histology independently. According to the hospital's internal review and ethics committee, all samples were collected with informed consent.

#### Group 2 for QPCR

84 cervical tumors were obtained from our department, between January 2002 and June 2011. None of them received any treatment before surgery. All patients have been confirmed HPV infection. Three patients without lesion on cervix were chosen as normal controls.

### MiRNAs real time polymerase chain reaction array

After isolation total RNA, Universal cDNA synthesis kit (Exiqon) was used. MiRNAs were detected by the miRCURY LNA™ Universal real time microRNA polymerase chain reaction system (Exiqon, kangchen, China).

### Cell culture

HeLa and SiHa were purchased from China Academic Sinica Cell Repository, Shanghai, China. They were in MEM medium (Gibco, Los Angeles, CA, USA) with 10% fetal bovine serum in an incubator under humidity at 37℃with 5% CO_2_.

### Transient transfection

MiR-100-5p mimics purchased from Genepharma (Shanghai, China), were transient transfected into cells with Lipofectamine™ 2000 (Invitrogen). Mock-transfected condition was made by negative control mimics with fluorescence. All the cells were collected at 48 h post-transfection, and the levels of miR-100 were elevated by RT-PCR assay as follow. The sequences of miR-100-5p mimics and negative control were shown below:

- Mimics (forward 5′-AACCCGUAGAUCCGAACUUGUG-3′.
- Reverse 5′-CAAGUUCGGAUCUACGGGUUUU-3′);
- Negative control (forward 5′-UUCUCCGAACGUGUCACGUTT-3′.
- Reverse 5′-ACGUGACACGUUCGGAGAATT-3′);

### miRNA expression analysis and RT-PCR assay

The RNAs were collected from cells by Trizol extraction (Invitrogen), and cDNAs were acquired using an RNA reverse transcription amplification kit (Takara). RT-PCR assays were performed by SYBR Green Real-time PCR Universal Reagent (GenePharma Co.Ltd.) and analyzed by BIO-RAD fluorescence quantitative PCR machine. Primers were as follows:

- miR-100-5p (5′-AACCCGTAGATCCGAACTTGTG-3′);
- u6 (5′-ACGCAAATTCGTGAAGCGTT-3′);
- GAPDH (forward 5′-TGGAAATGACAGTGAAGCACCTC-3′.
- reverse 5′-TCGTTCATGCACTCGCTGAAG-3′);

U6 was used as control for miRNA, GAPDH was used as control for mRNA. Both of them was calculated with the 2^−ΔΔCT^ method.

### Proliferation assay

After cultured (1 × 10^4^ cells/well) in separate 96-well plates for 24 h, cells were transfected with miR-100-5p mimics. At day 1, day 2, day 3, day 4 after transfection, cells activation were evaluated by Cell Counting Kit-8 assay, the absorbance were read at 450 nm.

### Tumor xenografts

Siha cells (2 × 10^6^) were transfected with miR-100 or Negative Control for 2 days following animal experiments institutional ethical guidelines. Cells were digested and resuspended in 200μL PBS.PBS with cells or without cells were injected into female BALB/c athymic nude mouse (4-week-old). Six nude mice were used. Mice were anesthetized with Isoflurane (Forene, Abbott GmbH & Co.KG, Wiesbaden, Germany) with 1 L*min^−1^ O2 flow for euthanasia.

### Bioinformatics

Two public algorithms MiRanda (http://www.microrna.org) and TargetScan (http://www.targetscan.org/) were chosen for predicting target genes. To avoid false positive predictions, only putative target genes predicted by both programs were accepted.

### Immunohistochemistry

After embedded by paraffin- and fixed with formalin, tissue was cut into 5 μm section. Deparaffinization, rehydration, quenche, and retrieve, incubation at 4 °C overnight with Anti-mTOR (phospho S2448) antibody (HRP) (ab196914) (Abcam).

### Establishment of radioresistant (RR) cell lines

Cells were exposed to two Gy 3 times, four Gy 3 times, six Gy 3 times, eight Gy 2 times and ten Gy 2 times at 300 cGy/min with a linear accelerator (SIMEN, Germany). [17, 18].

### 5-aza-2-Deoxycytidine treatment

HeLa and SiHa cells(3 × 10^5^cells/well) were cultured in 6-well plates overnight, then 10 μM DAC(5-aza-2-deoxycytidine**)** or 10 μM DMSO (dimethyl sulfoxide) was separately added to cells per 24 h. Cells were harvested after 5 days, and RNAs were extracted for detecting the level of mir-100 as mentioned above.

### Bisulfite sequencing PCR (BSP)

Following extraction (QIAamp DNA Mini Kit), Genomic DNA was subjected to EpiTect® Plus DNA Bisulfite Kit. After purification, modified DNA was chosen as nested PCR reactions template. The second-round PCR products were subjected to electrophoresis on agarose gels, excised, and inserted into the TOPO cloning vector (Invitrogen, K4600-01). Clones were used for DNA sequencing randomly.

Outer primers, 5′-ATTCGAATTTAGTGGAATTAGAATC-3′ (forward) and 5′-AACCTACAACAACAACAACAACG-3′(reverse);

Nested primers, 5′-TTAGTAATTTTAGGTTAGAGGGTTATCG-3′ (forward) and 5′-ACTCCAAAAACCCATAACTAACCG-3′ (reverse).

### Western blotting

Protein was extracted from cells with ice-cold radioimmunoprecipitation lysis solution, then centrifugated to remove cell debris. N-cadherin (ab18203) and E-cadherin (ab194982) from Abcam (Cambridge, MA, USA) were chosen as primary antibodies and HRP‐conjugated secondary antibody was purchased from Sangon (Shanghai, China).GAPDH and β-actin were employed as loading controls.

### CircRNA expression profiles in CC

We enriched the contained circRNAs and digested them with RNase A, then reversely transcribed them to RNA by fluorescent reagents and random primers. We used Human circRNA Arrays (8 × 15 K, Arraystar, Rockville, MD, USA) to determine the circRNA profile. Those circRNAs with fold change ≥ 2 were identified as differentially expression circRNAs. The data were analyzed by using R software and Arraystar program (Arraystar).

### Biotin-coupled probe pull-down assay

Hela and Siha cells were transfected by biotinylated miR-100-5p mimics or its mutant (Genepharma, Suzhou, China). Then cells underwent harvest, lysis, sonicate, and incubation with magnetic beads at 4℃ overnight. The RNA mix bound to the magnetic beads was eluted and treated with Trizol for further qRT-PCR.

### Florescent in situ hybridization

Fluorescent in situ hybridization probes for circCASC15, miR-100, and 18S RNA were designed and synthesized by Synbio Technologies (Suzhou, China). 18S RNA was used as a positive control. The signals of the probes were examined by the Florescent in Situ Hybridization Kit (RiboBio, Guangzhou, China).

### Data analysis

All data were based on three independent replicates. Statistical analyses were analyzed by SPSS 13.0. Data were presented as the mean values ± standard error. *P* < 0.05 was considered statistically significant.

## Results

### MiR-100 is down-regulated in radioresistant human cervical cancer

**Fig. 1:**
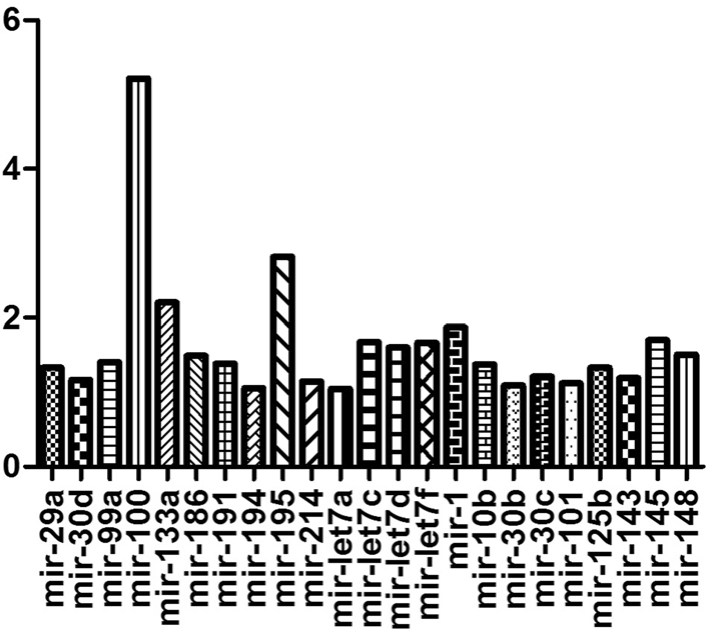
Foid changes of down-regulated miRNAs real time polymerase chain reaction array of radioresistant cervical cancer

To investigate miRNAs differential expressions in human radioresistant cervical cancer and to seek their roles in radioresistance, we performed miRNA microarray profiling analysis. At last we confirmed a set of miRNAs that are down-regulated in invasive cervical cancer. The most deregulated miRNA was miR-100 at 5 folds (*P* = 0.028) (Fig. 1).

### MiR-100 is correlated with poor clinical outcomes in cervical cancer

**Fig. 2:**
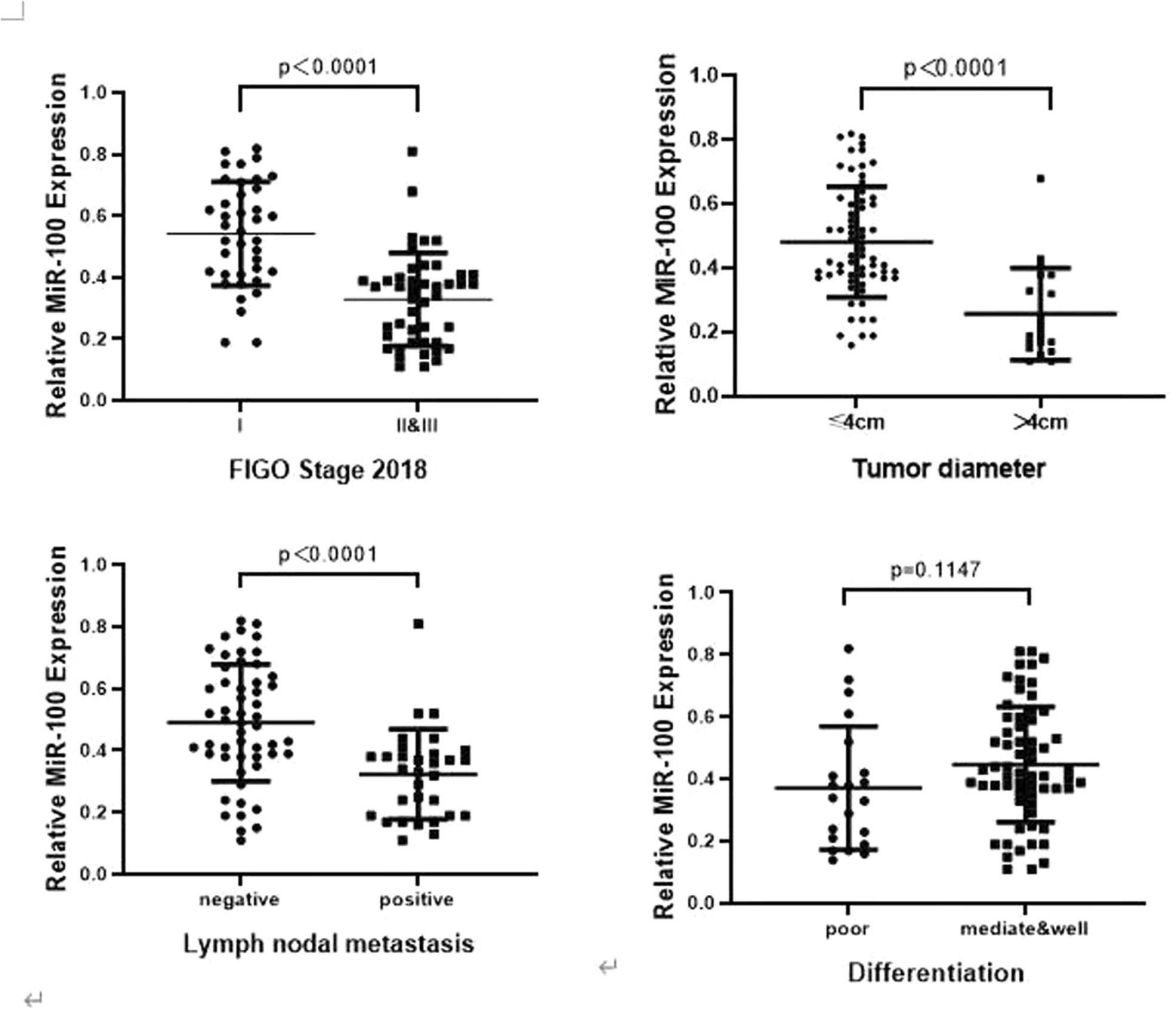
Low level of miR-100 is correlated with poor clinical outcomes in tumor size, lymph node status, TNM stage disease-free survival

The expression of miR-100 in 84 samples of cervical cancer and 3 normal cervical tissues from hysterectomy including uterine myoma or prolapse were tested by PCR. The average expression of miR-100 in cancer samples was lower than normal samples. We found that miR-100 expression was correlated with tumor size (*P* < 0.001), lymph node (LN) status (*P* < 0.001) and TNM stage (*P* < 0.001) (Fig. 2).

### MiR-100 inhibits invasion and reduces EMT

**Fig. 3:**
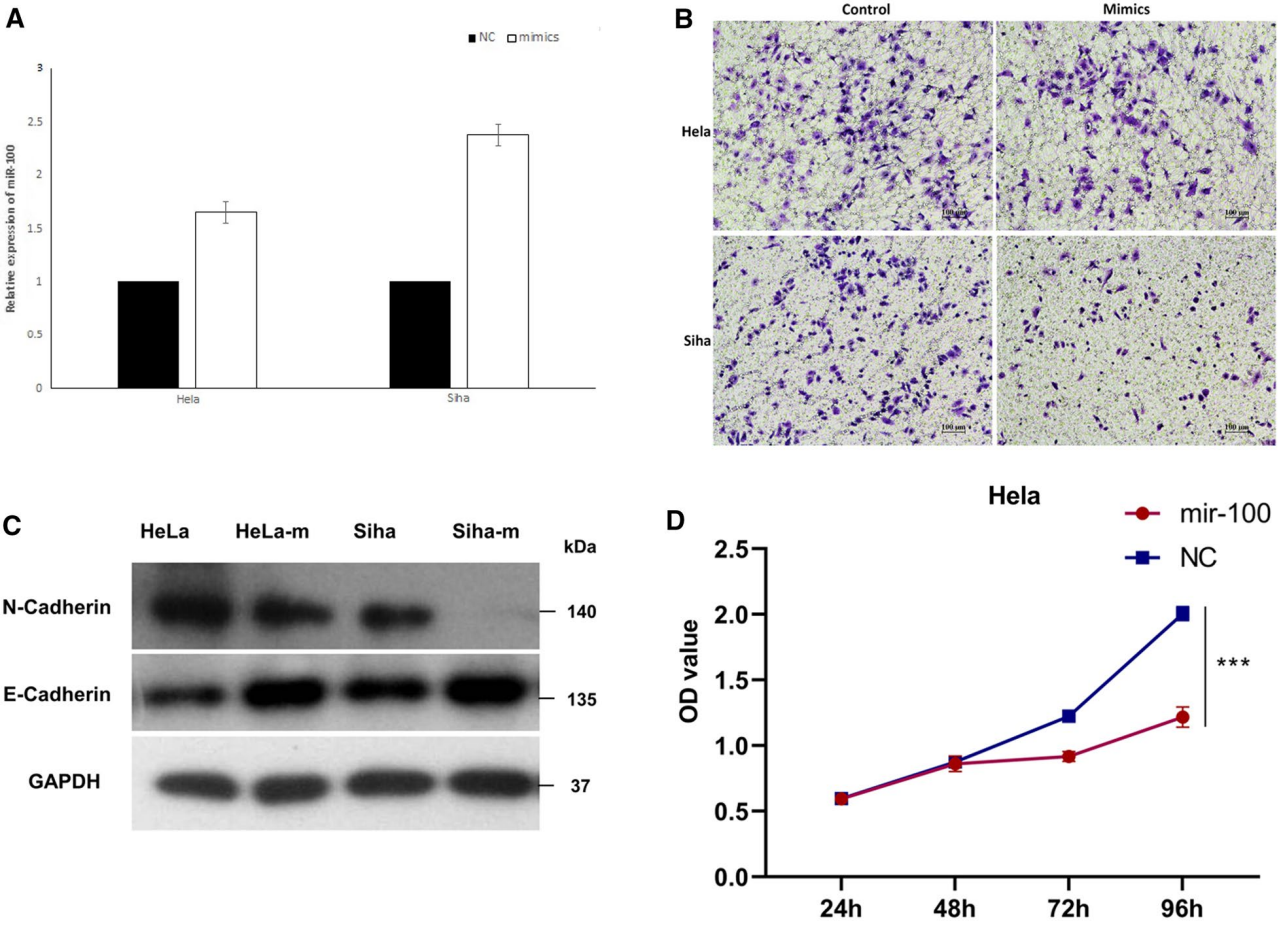

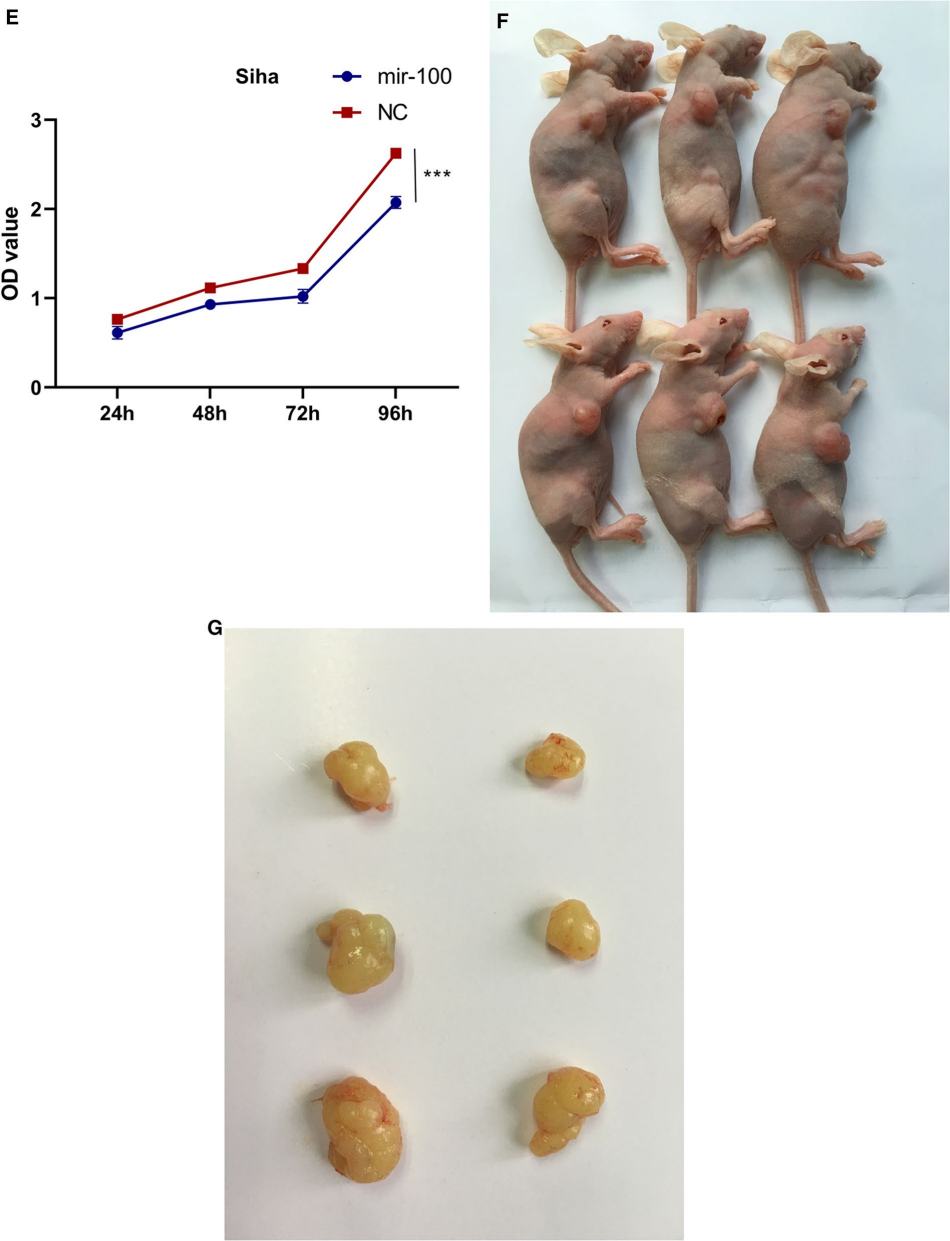
**A** MiR-100 mimics was used to up-regulate its expressions in Hela and Siha. **B** Cell invasiveness was tested by transwell assay in 24-well-plate which illustrated that miR-100 could inhibit invasion in Hela and Siha cells. **C** Western Blot showed the expression of N-cadherine and E-cadherine. **D** Proliferation was measured by CCK8 of Hela. **E** Proliferation of Siha. **F**, **G** MiR-100 inhibited tumorigenesis of SCC xenografts

Since the fact that miR-100 was decreased at fivefolds in invasive cervical cancer according to the array, miR-100 mimics was used to up-regulate its expressions in Hela and Siha (Fig. 3A). Cell invasiveness was tested by transwell assay in 24-well-plate which illustrated that miR-100 could inhibit invasion at Hela and Siha cells (Fig. 3B). Wondering whether the inhibition of invasiveness was mediated via EMT, western Blot was performed. As expected, up-regulation of miR-100 reduced EMT in protein levels (Fig. 3C).

### MiR-100 inhibits proliferation in cervical cancer

The observed down-regulation of miR-100 in human invasive cervical cancer tissues prompted us to detect whether it would be a tumor suppressor. Proliferation was measured by CCK8 kit after cells were transfected miR-100 mimics for 24, 48, 72 and 96h. No significant difference of proliferation of Hela (Fig. 3D) and Siha (Fig. 3E) were observed at the point of 24h after transfection. While at the other time points, proliferations were inhibited in cells treated with mimics.

### MiR-100 inhibits tumorigenesis in SCC xenografts

Three mice were injected cells transfected with miR-100, while others injected without transfection. Compared to control group, the volume of tumor was reduced (Fig. 3F and G).

### MiR-100 targets mTOR

**Fig. 4:**
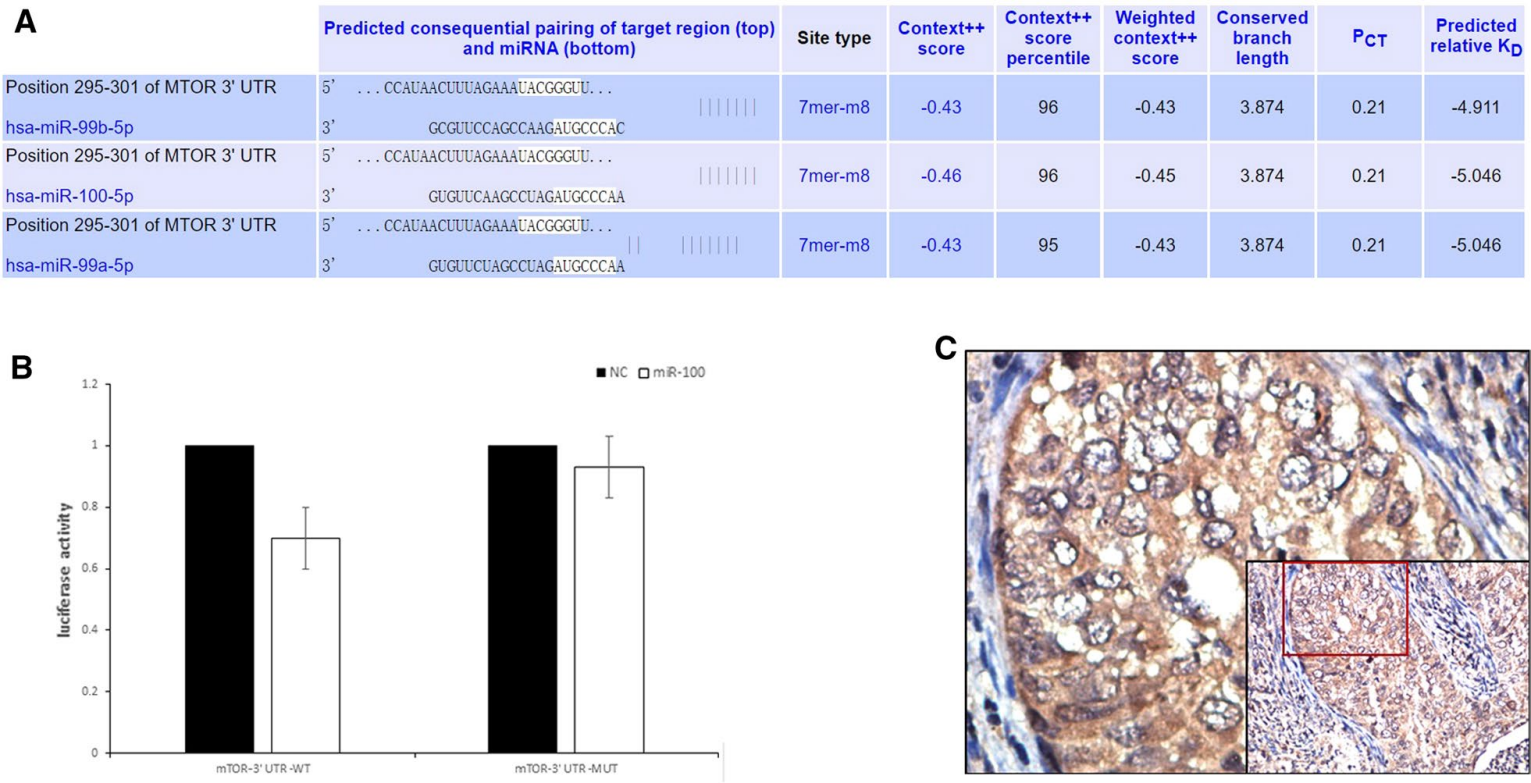
MTOR was a direct target of miR-100. **A** Putative miR-100-binding sequence in the 3′UTR of mTOR mRNA. **B** Luciferase activity on the presence of both wild-type mTOR 3′UTR or mutant and miR-100 was compared with those of the controls. *P < 0.05. **C** The expression of p-mTOR on cervical cancer was significantly higher than normal cervical

MiRanda and TargetScan predicted mTOR to be a target of miR-100.According to sequence analysis, mTOR was found contained a putative binding site of miR-100 (Fig. 4A). To verify whether mTOR was the target of miR-100, a wild-type mTOR 3'UTR fragment was cloned downstream of the firefly luciferase reporter gene. When pc3-miR-100 was co-transfected, the relative luciferase activity of the reporter gene containing the wild-type 3'UTR was inhibited significantly (Fig. 4B). And the expression of p-mTOR on cervical cancer was significantly higher than normal cervical tissue (Fig. 4C).

### MiR-100 was provoked by 5-Aza-CdR in HeLa and SiHa

**Fig. 5:**
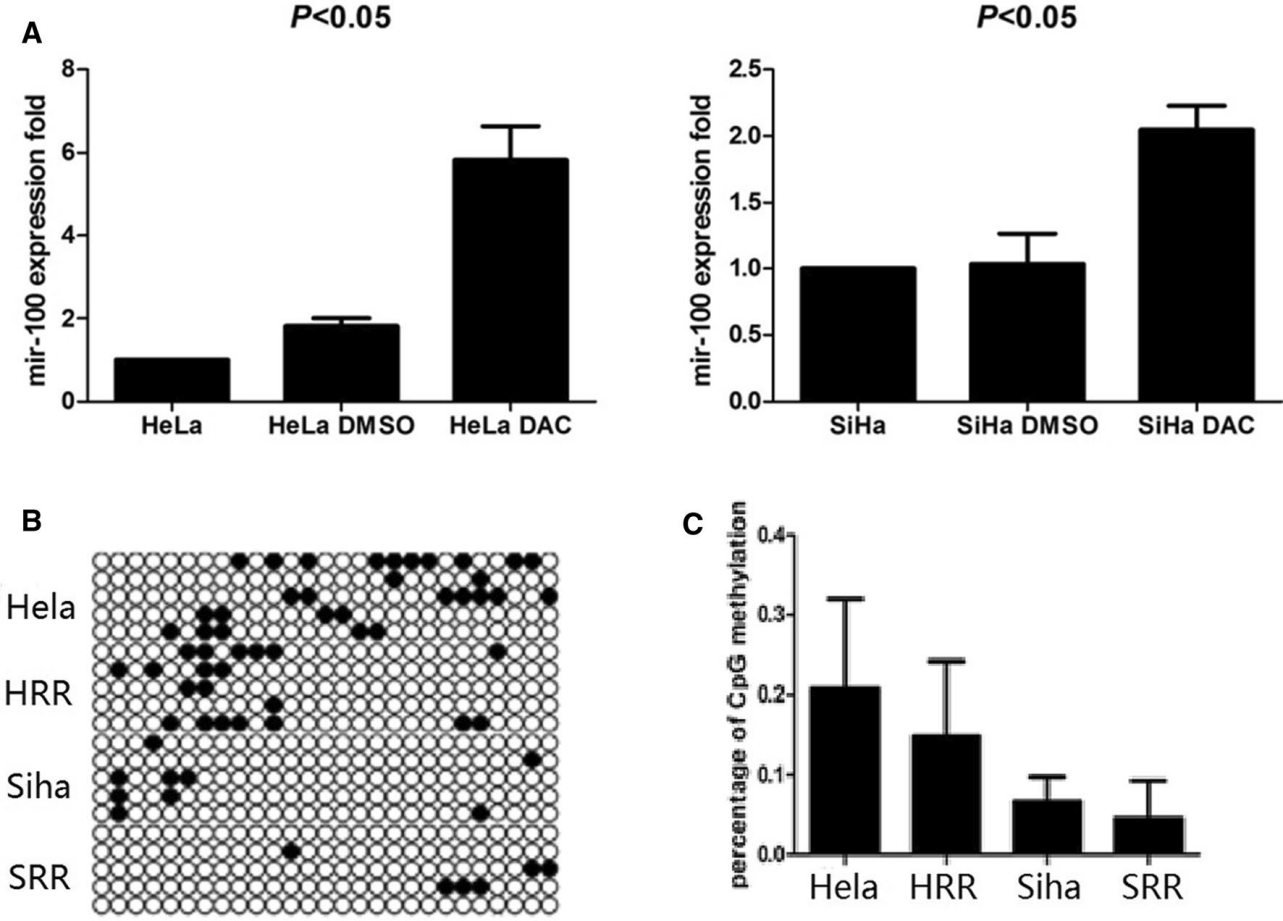
The methylation of miR-100HG in HeLa, HRR, SiHa and SRR. **A** 5-Aza-CdR provoked mir-100 in HeLa and SiHa. **B** Bisulfite sequencing PCR. Open circles: unmethylated CpG sites; solid black circles: methylated CpG sites. **C** The percentage of CpG methylation of the MIR-100HG in HeLa, HRR, SiHa and SRR

We applied 10 μM 5-Aza-CdR to HeLa and SiHa, to evaluate the effects on miR-100. As shown in Fig. 5A the treatment significantly up-regulated the level of miR-100 in both cell lines, suggesting a possible role of methylation in the regulation of miR-100 during the process of radiotherapy (*P* < 0.05).

### The level of miR-100 regulated by radiotherapy may not be mediated by miR-100HG

Bisulfite sequencing PCR was used to elevate the methylation of miR-100HG in HeLa, HRR, SiHa and SRR (Fig. 5B). The percentage of CpG methylation was 0.207 ± 0.050, 0.148 ± 0.042, 0.067 ± 0.014, 0.045 ± 0.021, respectively Fig. 5C. As shown in Fig. 5C, the methylation of miR-100HG decreased after radiotherapy, though there was no statistical significance.

### CiRcCASC15 may sponge miR-100 in ceRNA manner

**Fig. 6:**
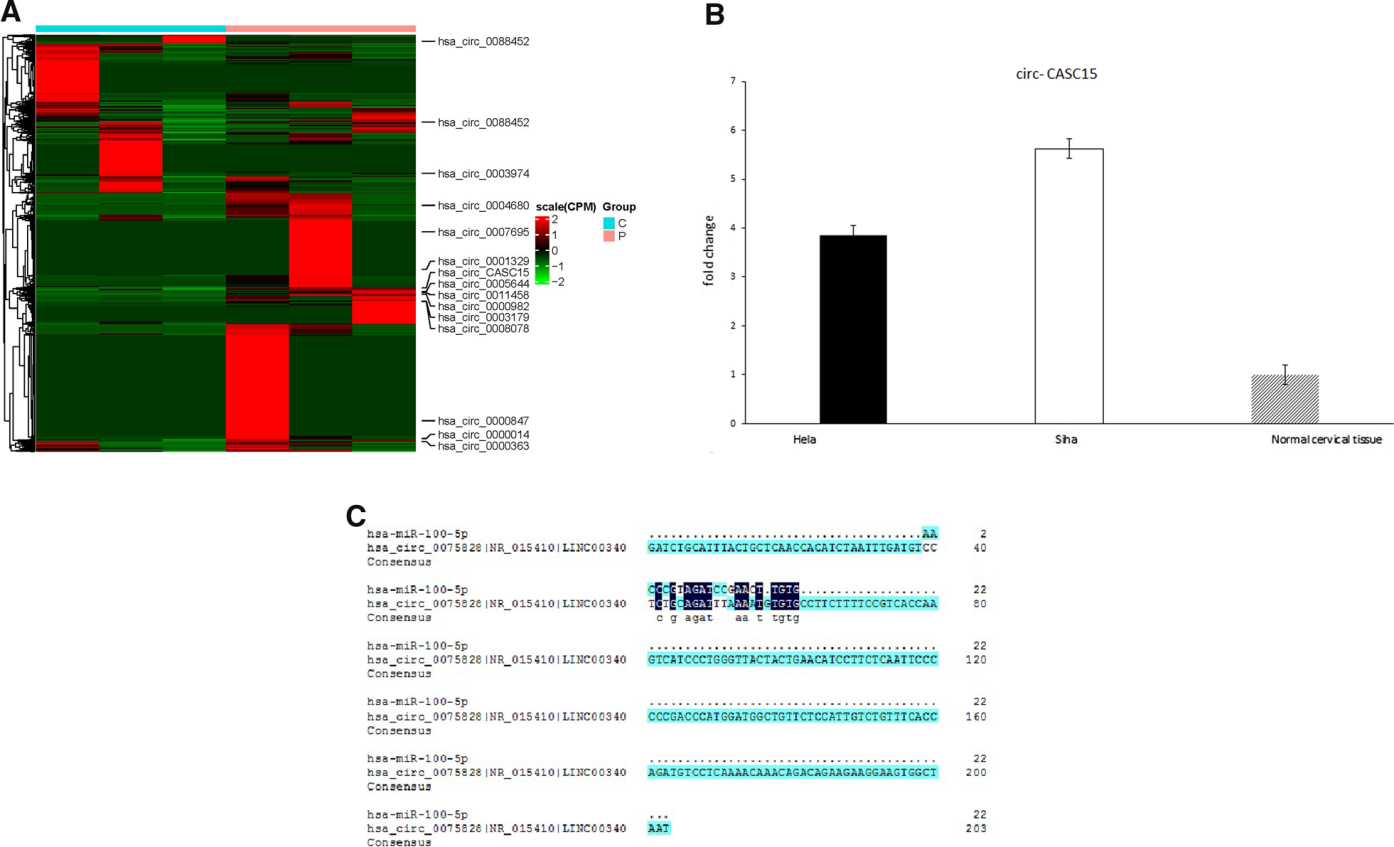

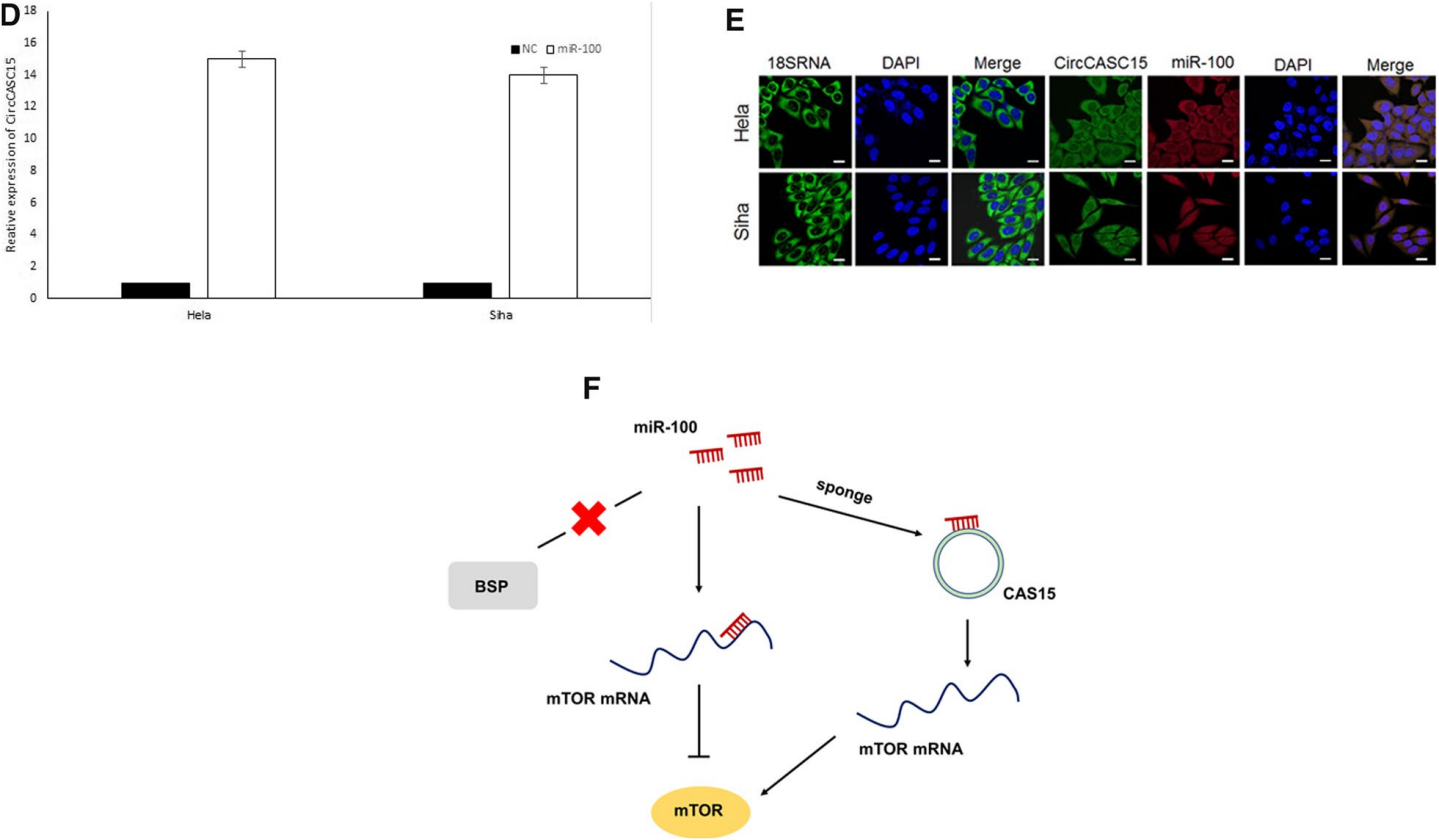
**A** The cluster heat map demonstrates the differentially expressed circRNAs over twofold change. **B** The expression of circCASC15 in CC was upregulated. **C** The predicted binding sites between circCASC15 and miRNA-100. **D** Pull-down assay for biotin labeled miRNA was used to evaluate binding properties between miR-100-5p and circCASC15 in Hela and Siha. **E** CircCasc15 and miR-100 were found mainly expressed in cytoplasm. **F** Graphical abstract

Three cervical cancer tissues and three normal cervical tissues were used for circRNAs expression profiling. The scatter and volcano plots demonstrated the result (Fig. 6A). The cluster heat map showed circRNAs that expressed over two fold change. QPCR was performed to verify the microarray results. Consistent with the microarray, qPCR showed that circCASC15 was increased in Hela and Siha cells compared to normal cervical tissues (Fig. 6B**)**.

With bioinformatic prediction, miR-100-5p was predicted as the sponge target of has-circ-CASC15 (Fig. 6C). To find the regulatory mechanism of miR-100, RNA pulldown assay was performed. CircCASC15 was found to be combined with miR-100 (Fig. 6D). CircCasc15 and miR-100 were found mainly expressed in cytoplasm. Both in our microarray analysis and tissues validation, circCASC15 was downregulated (Fig. 6E).

## Discussion

In the assay of breast cancer, the increase of miR-100 in the radio-resistant cell lines was detected [19]. While miR-100 was considered to be a suppressor gene, which used to decrease in cervical cancer. In this assay, its expression suggested a potential role in sensitivity of radiotherapy. In oral cancer, the increase of miR-100 inhibited FGFBP1 and FGFR3, which over-expressed in radio-resistant cells [8], indirectly corroborate our hypothesis.

It is recorded that DNA methylation may play an important role in silencing microRNA in tumors. A meta-analysis of 122 microRNAs showed that microRNAs regulated by methylation were mainly located in chromosome 1, 7, 11, 14 and 19 [20]. As miR-100 located in chromosome 11, we inferred that methylation may contribute to the regulation of it. Thereby we detected the effects of 5-Aza-CdR on HeLa and SiHa. With the treatment, the expressions of miR-100 were significantly induced. Our evidence linked methylation to irradiation on the increase of miR-100 in HeLa and SiHa, suggesting the level of methylation may have changed in the process of radiotherapy.

In breast cancer, miR-100 has been proved to be regulated by the methylation of miR100HG, a host gene, where the miR-100 gene embedded [21]. Furthermore, we elevated the methylation of MiR-100HG in SiHa, SRR, HeLa and HRR to validate our hypothesis. The Bisulfite sequencing PCR showed that the percentage of CpG methylation slightly decreased in HRR and SRR, compared to Hela and SiHa. Accordingly, the level of miR-100 regulated by radiotherapy may not be directly mediated by the methylation of miR100HG. As the loss of chromosome 11 also can lead to the low expression of miR-100 [22], the regulation of miR-100 remains to further research.

It’s widely shared that miRNAs could bind to 3’UTR leading mRNA cleavage or translation inhibition which negatively regulate downstream mRNA and transcription factors [23]. While as a single miRNA can regulate sorts of mRNAs and in return one individual mRNA could be controlled by several miRNAs. Nevertheless, genes regulated by one miRNA often have similar functions. The identified targets of miR-100 include mTOR [7, 23]. They both act as cell cycle activate genes which enable and allow cells entry into mitosis [24–26].

MTOR is a protein kinase belonging to PI3K family [27]. As the core component of many different complexes, mTOR plays a key role in a variety of biological processes [27, 28]. Up-regulating mTOR signaling can promote tumor growth and progression through a variety of mechanisms [29]. One study has revealed increased expression of mTOR in cervical cancer [30]. In all cases, SCCs showed high translocation of p-mTOR and p-p70S6K in nuclear [31]. Faried et al. found activated AKT and mTOR related with poorer prognosis. The same authors [32] also investigated the expression of phosphorylated mTOR was independent and was a significant prognostic marker in cervical adenocarcinoma. In addition, the expression of p-mTOR can be used as a marker to predict chemotherapy response and survival of CC [33]. Moreover, Kim et al. [34] have also reported that p-mTOR was associated with poor response to radiotherapy. They [32] also independently studied the expression of p-mTOR as an important prognostic marker of cervical adenocarcinoma. In addition, the expression of phosphorylated mTOR can be used to predict the chemotherapy response and survival [33]. The cytoplasmic expression of p-mTOR was also related to the adverse reactions of radiotherapy [34].

CircCASC15 was also named hsa_circ_0075828. The circCASC15, generated from CASC15 gene, was 204 nucleotides in length. Divergent primers were designed and confirmed by Sanger sequencing. MRE (miRNA response element) has been proved existing in circRNA and acts as a ceRNA sponge or transcription regulator. There has been no report about circCASC15 in cervical cancer before our study.

In conclusion, circCASC15-miR-100-mTOR might influence the EMT of cervical cancer. And this loop will be the target of radioresistance in the future.

## Data Availability

The datasets generated and/or analysed during the current study are available from the corresponding author on reasonable request.

## Abbreviations

circRNA: Circular RNA
ceRNAs: Competitive endogenous RNAs
CC: Cervical cancer
NC: Negative control
RR: Radioresistant
EMT: Epithelial-Mesenchymal Transition
MRE: MiRNA response element
